# A comparative evaluation between cheiloscopic patterns and canine relationship in permanent dentition

**DOI:** 10.4317/jced.62752

**Published:** 2025-06-01

**Authors:** Elanthendral Saravanan, Vignesh Ravindran, Abirami Arthanari

**Affiliations:** 1 Post graduate, Dept of Pediatrc and Preventive Dentistry, Saveetha Dental College & Hospitals, Saveetha Institute of Medical and Technical Sciences, Saveetha University, Chennai, Tamilnadu, India; 2 Associate Professor, Dept of Pediatrc and Preventive Dentistry, Saveetha Dental College & Hospitals, Saveetha Institute of Medical and Technical Sciences, Saveetha University, Chennai, Tamilnadu, India; 3 Lecturer, Dept of Forensic Odontology, Saveetha Dental College & Hospitals, Saveetha Institute of Medical and Technical Sciences, Saveetha University, Chennai, Tamilnadu, India

## Abstract

**Background:**

The precise determination of occlusal relationships, particularly the alignment of maxillary and mandibular canines, is fundamental to orthodontic diagnosis and treatment planning. Cheiloscopy, the study of lip print patterns, has gained prominence in forensic odontology and is hypothesized to have potential relevance in orthodontics. This study aims to evaluate the relationship between cheiloscopic patterns and canine relationships in permanent dentition to assess its diagnostic significance.

**Material and Methods:**

A cross-sectional observational study was conducted on 300 adolescents (aged 14–16 years) from Chennai. Canine relationships were classified as Class I, Class II, or Class III based on standard intraoral examination protocols. Lip prints were collected using the lipstick-cellophane transfer technique and analyzed based on the Suzuki and Tsuchihashi classification system. Statistical analysis, including the Chi-Square test and Fisher’s Exact Test, was performed using SPSS software (version 22.0), with a significance threshold of *p* < 0.05.

**Results:**

Type II (branched) lip prints were the most prevalent across all canine relationships (71.3%), followed by Type IV (reticular) (17.7%). A significant gender-based difference was observed in Class III canine relationships (p = 0.003), where males predominantly exhibited Type II patterns (86.7%), while females showed a higher frequency of Type IV patterns (36.4%). No significant gender differences were noted in Class I and Class II relationships.

**Conclusions:**

Cheiloscopic patterns demonstrate notable variations across different canine relationships, with significant gender-based differences in Class III relationships. These findings suggest that cheiloscopy may serve as a non-invasive adjunct in orthodontic diagnosis and forensic odontology.

** Key words:**Cheiloscopy, canine relationships, orthodontic diagnosis, lip print patterns, forensic odontology.

## Introduction

The precise determination of occlusal relationships in permanent dentition, particularly concerning the interarch alignment of maxillary and mandibular canines, is fundamental to orthodontic diagnosis and treatment planning. The morphological characteristics of the occlusal scheme exhibit significant variability, influenced by genetic, environmental, and ethnic factors. Epidemiological studies have underscored the prognostic significance of occlusal traits in the primary dentition, as they serve as reliable predictors of future malocclusions in the permanent dentition (1). Early identification of deviations from normal occlusal patterns allows for timely interceptive orthodontic measures, reducing the complexity of future orthodontic interventions and improving long-term stability.

In parallel to these occlusal considerations, cheiloscopy, the study of lip print patterns, has gained attention in forensic odontology as a potential diagnostic and predictive tool. The intricate sulci labiorum rubrorum, described by Fischer in 1902 and later classified by Suzuki and Tsuchihashi, exhibit unique morphological patterns that remain unchanged throughout life (2). These patterns, formed during embryogenesis, are genetically determined and resistant to environmental influences (3). Given the shared developmental origins of orofacial structures, including the maxillofacial skeleton, alveolus, and soft tissues, it has been hypothesized that cheiloscopic patterns may reflect underlying craniofacial growth patterns, including occlusal development.

If a definitive correlation between cheiloscopic patterns and canine relationships can be established, cheiloscopy could serve as a novel, non-invasive diagnostic adjunct in orthodontics (4). Given that lip print patterns are genetically determined and remain sTable throughout life, they may provide an early phenotypic marker of underlying craniofacial growth patterns, including occlusal development. By analyzing an individual’s cheiloscopic pattern, clinicians could potentially anticipate deviations in canine interarch relationships, allowing for early identification of malocclusion risks even before conventional clinical or radiographic signs become apparent (5). This predictive insight could facilitate timely interceptive orthodontic interventions, enabling practitioners to implement measures such as space maintenance, myofunctional therapy, or guided eruption strategies. Integrating cheiloscopy into orthodontic diagnostic protocols could therefore enhance patient-specific treatment planning, optimize long-term occlusal outcomes, and reduce the complexity of future orthodontic corrections.

Thus, the present study aims to comparatively evaluate the relationship between distinct cheiloscopic patterns and canine relationships in permanent dentition. By investigating this potential correlation, the study seeks to enhance the orthodontic applicability of cheiloscopy, expanding its role beyond forensic identification to serve as a predictive adjunct in orthodontic diagnosis and treatment planning.

## Material and Methods

- Study Design and Ethical Considerations

This cross-sectional observational study was conducted over a period of six months, from February 2024 to July 2024, within the Outpatient Department of Pediatric and Preventive Dentistry. Ethical approval was duly obtained from the Institutional Review Board (SRB/SDC/UG/Pedo/2024/03/067), ensuring compliance with ethical research protocols. The study objectives and procedural methodology were meticulously explained to the parents or legal guardians of all prospective participants, and written informed consent was secured prior to enrollment.

- Study Population and Eligibility Criteria

A total of 300 children, aged between 14 and 16 years, were recruited for the investigation. Participants were selected based on specific inclusion criteria, necessitating them to be native residents of Chennai, as substantiated by their birth certificates. Only those whose parents provided written informed consent were considered eligible for inclusion. Additionally, participants were required to exhibit a fully erupted permanent dentition, excluding third molars, with well-established occlusal relationships.

Conversely, individuals exhibiting uncooperative behavior or presenting with any form of lip pathology, including congenital anomalies such as cleft lip, acquired defects like burns, scars, or lacerations, or a history of chemical or thermal trauma were systematically excluded from the study. Additional exclusion criteria encompassed a prior history of orthodontic treatment, retained primary teeth or root remnants, extensive proximal caries, premature primary tooth extractions, and inconsistent canine relationships on either side of the dental arch.

- Determination of Canine Relationships

Two trained and calibrated examiners conducted an intraoral examination to classify canine relationships into three established categories: Class I, Class II, and Class III. A Class I canine relationship was determined when the cusp tip of the maxillary canine occluded precisely with the distal surface of the mandibular canine. A Class II canine relationship was characterized by the mesial positioning of the maxillary canine’s cusp tip relative to the distal surface of the mandibular canine, indicative of a more pronounced maxillary prominence. Conversely, a Class III canine relationship was identified when the cusp tip of the maxillary canine was positioned distal to the distal surface of the mandibular canine, suggestive of mandibular prognathism.

The assessment was meticulously performed using a mouth mirror under optimal illumination, ensuring accuracy and reliability in classification. The findings of both examiners were compared for inter-examiner agreement, and only those cases where complete concordance was achieved were included in the final analysis. Any instances of discrepancy between the examiners resulted in the exclusion of the respective participant from the study.

- Cheiloscopic Data Collection and Processing

Lip print acquisition was performed using the lipstick-cellophane transfer technique described by Sivapathasundaram *et al*., recognized for its clarity and reproducibility (6). Prior to obtaining the prints, the participants’ lips were cleansed with a sterile, moistened cotton pad and subsequently allowed to air dry. A matte lipstick formulation was applied using a single-use cotton applicator, ensuring uniform coverage across the vermilion zone. The upper lip was coated with one end of the applicator, while the lower lip was coated with the opposite end, thereby preventing cross-contamination. The disposable cotton swabs were discarded in strict adherence to aseptic protocols.

Following application, the participants were instructed to rub their lips together gently to ensure even distribution of the lipstick, after which they were asked to relax their lips in a natural resting position. A transparent cellophane sheet was then carefully positioned over the lips and held in place for a few seconds, allowing for the transfer of the lip print. The sheet was subsequently lifted and affixed onto a white paper backing to preserve the impression. In instances where the print was unclear or distorted, the process was repeated to ensure an optimal imprint. Residual lipstick was removed using a sterile wet tissue.

A forensic expert, blinded to the participants’ canine relationships, examined the lip prints under magnification to ensure an unbiased classification. Lip prints were categorized based on the widely accepted Suzuki and Tsuchihashi classification system (7), which delineates six distinct morphological patterns:

● Type I: Complete vertical grooves

● Type I’: Incomplete vertical grooves

● Type II: Branched grooves

● Type III: Intersecting grooves

● Type IV: Reticular or net-like grooves

● Type V: Indeterminate or irregular patterns

For standardization and to mitigate potential inter-individual variability, only the central 10 mm segment of the lower lip was selected for analysis, as per the methodology established in prior research by Sivapathasundaram *et al*. (8). The most predominant pattern observed within this defined region was recorded for each participant.

- Statistical Analysis

All collected data were systematically tabulated and subjected to statistical scrutiny. The distribution of canine relationships and cheiloscopic patterns was analyzed and compared across gender groups using the Chi-Square test. Additionally, Fisher’s Exact Test was employed in instances where the expected frequency within any given cell was found to be less than five. Statistical computations were executed using SPSS software (version 22.0, SPSS Inc., Chicago, IL, USA). A *p-value* of less than 0.05 was considered statistically significant, ensuring robust inferential conclusions regarding the correlation between cheiloscopic patterns and canine relationships.

## Results

- Demographic Characteristics and Distribution of Canine Relationships

The study cohort comprised 300 adolescents aged between 14 and 16 years, with a mean age of 15.31 ± 0.67 years. The participants were categorized based on their canine relationships into Class I (n = 174), Class II (n = 48), and Class III (n = 78). A detailed analysis of gender distribution within these classifications revealed that 44.8% of individuals with Class I canine relationships were female, while 55.2% were male. In contrast, among participants exhibiting Class II canine relationships, 52.1% were female and 47.9% were male, indicating a relatively balanced gender representation within this group. However, in Class III canine relationships, a noTable male predominance was observed, with 57.7% of the affected individuals being male, compared to 42.3% female, suggesting a potential sex-linked predisposition to this occlusal pattern.

- Distribution of Cheiloscopic Patterns Among Canine Relationships

The analysis of cheiloscopic patterns in relation to canine classifications revealed distinct distributions across Class I, Class II, and Class III canine relationships. A total of 214 participants (71.3%) exhibited Type II (branched) lip patterns, making it the most prevalent cheiloscopic pattern observed across all canine classifications. Among these, 122 individuals (70.1%) belonged to Class I canine relationships, while 34 (70.8%) and 58 (74.4%) corresponded to Class II and Class III canine relationships, respectively. This pattern demonstrated a consistent predominance across all canine classifications.

The Type IV (reticular) lip pattern was the second most frequently observed, recorded in 53 participants (17.7%). This pattern was more prevalent in Class II relationships, accounting for 11 individuals (22.9%), followed by 14 individuals (17.9%) in Class III and 28 individuals (16.1%) in Class I.

The Type I (complete vertical) pattern was identified in 16 participants (5.3%), predominantly among those with a Class I canine relationship (13 participants, 7.5%). Notably, no cases of this pattern were observed among individuals with Class II canines, whereas only 3 participants (3.8%) in the Class III category exhibited this pattern. The Type I’ (incomplete vertical) pattern was the least common, found in only 3 participants (1.0%), all of whom had a Class I canine relationship.

The Type III (intersected) lip pattern was rare, observed in 3 participants (1.0%) overall, with only 2 cases (1.1%) in Class I and 1 case (1.3%) in Class III, while it was completely absent in Class II canine relationships. Similarly, the Type V (irregular or undetermined) pattern was noted in 11 participants (3.7%), with the highest proportion in Class II (3 participants, 6.3%), followed by 6 individuals (3.4%) in Class I and 2 individuals (2.6%) in Class III.

- Gender-Based Distribution of Cheiloscopic Patterns Across Canine Relationships

A comprehensive analysis of gender-based variations in cheiloscopic patterns among different canine classifications was conducted, revealing distinct distribution trends. While certain patterns exhibited a higher prevalence in one gender over the other, the overall differences in Class I and Class II canine relationships were not statistically significant. However, in Class III canine relationships, a significant gender disparity was observed (*p* = 0.003), indicating a potential gender influence on the distribution of cheiloscopic patterns within this subgroup.

- Class I Canine Relationship

Among individuals exhibiting a Class I canine relationship, Type II (branched) lip prints were the most predominant in both males (71.9%) and females (67.9%), reinforcing the high prevalence of this pattern irrespective of gender. Type IV (reticular) lip prints were the second most common, observed in 12.5% of males and 20.5% of females, suggesting a slightly greater prevalence in females. Type I (complete vertical) lip patterns were recorded in 8.3% of males and 6.4% of females, whereas the Type I’ (incomplete vertical) pattern was exclusively present in males (3.1%), with no occurrences in females. Type III (intersected) patterns were observed in only 1 male (1.0%) and 1 female (1.3%), while the Type V (irregular or undetermined) pattern showed a minimal presence, with 3.1% in males and 3.8% in females. The overall statistical comparison of gender distribution within Class I was not significant (*p* = 0.473), suggesting no substantial gender-based differentiation in lip print patterns within this occlusal category.

- Class II Canine Relationship

Similar trends were observed in individuals classified under the Class II canine relationship, where Type II (branched) lip prints remained the predominant pattern, observed in 73.9% of males and 68.0% of females. The Type IV (reticular) pattern followed in frequency, with 21.7% of males and 24.0% of females, displaying a comparable distribution between genders. The Type V (irregular) pattern was relatively rare, recorded in 4.3% of males and 8.0% of females. Notably, Type I, Type I’, and Type III patterns were entirely absent in both genders for this canine classification. Statistical analysis demonstrated no significant gender-based variation in the distribution of cheiloscopic patterns among individuals with Class II canine relationships (*p* = 0.843).

- Class III Canine Relationship

A notable gender disparity emerged within the Class III canine relationship category (*p* = 0.003), highlighting a statistically significant difference in lip print pattern distribution between males and females. The Type II (branched) pattern remained predominant but exhibited a pronounced gender variation, occurring in 86.7% of males compared to 57.6% of females, indicating a higher affinity of this pattern in males with Class III canine relationships. Conversely, the Type IV (reticular) pattern demonstrated a strikingly higher prevalence in females (36.4%) compared to males (4.4%), suggesting a potential gender-specific association with this pattern.

The Type I (complete vertical) lip pattern was observed in 2.2% of males and 6.1% of females, while the Type III (intersected) pattern was present in 2.2% of males but entirely absent in females. Interestingly, the Type V (irregular) pattern was recorded in 4.4% of males, with no occurrences in females, reinforcing the gender-based discrepancy within this category. The absence of the Type I’ (incomplete vertical) pattern in both males and females within the Class III canine classification further underscored the selective nature of lip print pattern distribution ( Table 1, Table 2).

## Discussion

The transition from primary to permanent dentition follows a meticulously regulated developmental sequence, wherein the primary dentition serves as a functional matrix guiding the eruption, alignment, and occlusal stability of the succeeding permanent teeth. Disruptions in this sequence—whether due to premature tooth loss, parafunctional habits, or pathological conditions such as extensive dental caries—can lead to spatial discrepancies, arch length discrepancies, and occlusal disharmony (9). Among permanent teeth, the canines play a pivotal role in maintaining occlusal equilibrium, guiding lateral mandibular movements, and preventing occlusal interferences. Any variation in their interarch relationship—classified as Class I, Class II, or Class III canine relationships—can have significant functional and esthetic implications, affecting both occlusal harmony and long-term stability (10).

The embryological parallels between palatal formation, alveolar development, and labial differentiation suggest a potential correlation between cheiloscopic patterns and occlusal characteristics, particularly in relation to canine interarch relationships. Previous studies have explored associations between lip print patterns and malocclusion, molar relationships, and various dental anomalies (11). However, the direct relationship between cheiloscopic patterns and canine relationships in permanent dentition remains an unexplored domain in orthodontic research. Given that canine guidance plays a crucial role in occlusal function and stability, identifying a correlation between lip print morphology and canine occlusion could offer valuable insights into early malocclusion risk assessment (12).

The present study aimed to evaluate the association between cheiloscopic patterns and different canine relationships in permanent dentition. The results demonstrated that the Type II (branched) pattern was the most predominant across all canine relationships, followed by the Type IV (reticular) pattern, which showed a higher frequency in Class II relationships. The Type I (complete vertical) pattern was more commonly observed in Class I, while Class III relationships exhibited a predominance of Type IV (reticular) and Type V (irregular) patterns, with an absence of Type I’ (incomplete vertical) patterns. Additionally, no statistically significant relationship was found between cheiloscopic patterns and different canine classifications, except for a significant gender-based difference in Class III, where males predominantly exhibited Type II (branched) patterns and females exhibited an increased frequency of Type IV (reticular) patterns. These findings indicate a possible correlation between cheiloscopic features and occlusal development, warranting further exploration into the genetic and developmental basis of these associations.

The present study did not yield a significant association between cheiloscopic pattern distribution and canine relationships, as reflected by a *p-value* of 0.538. This suggests that while certain trends in lip print distribution were evident among different canine classifications, these variations were not statistically significant within the scope of the study population. However, the predominance of Type II (branched) and Type IV (reticular) patterns across all canine classes suggests a potential underlying association that warrants further exploration with a larger sample size and advanced multivariate statistical models.

While gender-based differences in cheiloscopic pattern distribution did not reach statistical significance in Class I (*p* = 0.473) and Class II (*p* = 0.843) canine relationships, the significant variation observed in Class III (*p* = 0.003) suggests a potential gender influence in lip print pattern expression within this occlusal category. These findings indicate that cheiloscopy may hold diagnostic relevance in orthodontic evaluations, particularly in identifying Class III canine relationships with gender-specific pattern variations.

The methodology employed in the study was chosen to ensure high accuracy, reproducibility, and clinical relevance. The lip print acquisition technique followed the established lipstick-cellophane method, which has been widely used due to its ease of application, non-invasiveness, and high precision in capturing cheiloscopic details (13). To minimize subjective bias, the lip prints were analyzed by a blinded forensic expert, and canine classification was determined by two calibrated examiners to ensure inter-examiner reliability. The central 10 mm segment of the lower lip was selected for analysis, in accordance with previous studies, as this region has been found to exhibit the most sTable and reproducible patterns (14).

The findings of this study align with previous research while also highlighting novel aspects of cheiloscopic pattern distribution in different occlusal relationships. Vignesh *et al*. (2019) also reported the predominance of Type II (branched) patterns, which were equally distributed among different molar relationships (4). However, our study demonstrated a noTable increase in Type IV (reticular) patterns in Class III malocclusions, particularly among females, a finding that was statistically significant (*p* < 0.003). In contrast, Anuradha (2020) reported no statistically significant correlation between cheiloscopic patterns and skeletal malocclusion (*p* = 0.8), which diverges from our findings in Class III relationships (15). This discrepancy may be attributed to differences in study populations, sample sizes, or methods of skeletal classification.

Similarly, Vignesh *et al*. (2018) found a predominance of Type II (branched) patterns in children with primary dentition, with a significant presence of Type IV and Type I patterns in Class II relationships, supporting our observations (8). However, their study noted a higher prevalence of Type V (irregular) patterns in Class III relationships, while our study found a predominance of Type IV (reticular) patterns in females and Type II (branched) patterns in males within the same occlusal category. This suggests that gender-specific variations may play a role in cheiloscopic pattern distribution, particularly in Class III occlusions, which has not been extensively explored in previous studies.

A major difference between the current study and Vignesh (2024) is the method of dental assessment, as the latter focused on dermatoglyphic patterns in primary dentition rather than permanent canine relationships (16). Their results indicated no significant correlation between dermatoglyphics and occlusal patterns, reinforcing the notion that cheiloscopy might serve as a more reliable biomarker for occlusal classification than dermatoglyphics.

This study introduces several novel aspects to the field of forensic odontology and orthodontic diagnosis. Unlike previous studies, our research specifically examines the gender-based variations in cheiloscopic pattern distribution within different canine relationships. The statistically significant difference in Class III relationships suggests a possible sex-linked influence on lip print expression, which has not been extensively investigated in orthodontic literature. Additionally, this study provides a robust dataset on cheiloscopic trends in permanent dentition, adding valuable epidemiological insight into the interplay between lip morphology and occlusal development. The use of a blinded forensic expert for cheiloscopic analysis, combined with calibrated orthodontic evaluations, enhances the study’s methodological rigor and reliability.

The findings of this study carry important clinical implications for orthodontists, forensic dentists, and pediatric dentists. Given the observed associations between cheiloscopic patterns and canine relationships, cheiloscopy could serve as an adjunctive diagnostic tool in orthodontic assessment and treatment planning. Early identification of high-risk occlusal patterns based on lip print analysis may facilitate predictive orthodontic interventions, particularly in cases prone to Class III malocclusion. Additionally, the gender-based discrepancies in lip print distribution suggest that sex-linked factors may influence occlusal development, which could be considered when formulating personalized orthodontic treatment strategies (17).

From a forensic perspective, cheiloscopy has been extensively utilized in human identification. The results of this study reinforce its potential application in forensic odontology, particularly in reconstructing occlusal characteristics from lip print evidence in medico-legal investigations. The findings also hold relevance in craniofacial growth studies, as they provide insight into the relationship between perioral soft tissues and occlusal variations, which could further aid in understanding the genetic determinants of malocclusion (18).

This study has certain limitations, primarily its cross-sectional design, which limits the ability to establish causality between cheiloscopic patterns and occlusal development; a longitudinal study would provide stronger evidence. Additionally, as the study was conducted in a single geographical region (Chennai), the findings may not be generalizable to diverse populations, necessitating multicentric studies. Variations in lip elasticity and pressure during imprinting may have introduced minor inconsistencies, which could be minimized using advanced digital analysis techniques. Furthermore, while a significant gender-based difference in Class III relationships was observed, the underlying genetic or developmental mechanisms remain unclear, highlighting the need for genetic and molecular research to better understand the interaction between perioral soft tissues and occlusal development.

## Conclusions

This study provides compelling evidence that cheiloscopic patterns exhibit notable variations across different canine relationships in permanent dentition, with significant gender-based differences in Class III canine relationship. The Type II (branched) pattern emerged as the most prevalent across all the groups, while Type IV (reticular) patterns were more frequent in Class II and Class III relationships, particularly among females. These findings suggest that cheiloscopy may serve as a valuable adjunct in orthodontic diagnosis and forensic odontology. Further research incorporating longitudinal and genetic studies is warranted to explore the developmental underpinnings of these observed associations.

## Figures and Tables

**Table 1:** Cheiloscopic distribution among different classes of canines in permanent dentition.

Cheiloscopic pattern	Class-I		Class-II		Class-III		Total		P value
	N	%	N	%	N	%	N	%	
I	13	7.5	0	0.0	3	3.8	16	5.3	0.538
I'	3	1.7	0	0.0	0	0.0	3	1.0	
II	122	70.1	34	70.8	58	74.4	214	71.3	
III	2	1.1	0	0.0	1	1.3	3	1.0	
IV	28	16.1	11	22.9	14	17.9	53	17.7	
V	6	3.4	3	6.3	2	2.6	11	3.7	

**Table 2:** Gender comparison on distribution of cheiloscopic pattern among different classes of canines in permanent dentition.

Canine	Pattern	Gender				P value
		Male		Female		
		N	%	N	%	
Class-I	I	8	8.3	5	6.4	0.473
	I'	3	3.1	0	0.0	
	II	69	71.9	53	67.9	
	III	1	1.0	1	1.3	
	IV	12	12.5	16	20.5	
	V	3	3.1	3	3.8	
Class-II	I	0	0.0	0	0.0	0.843
	I'	0	0.0	0	0.0	
	II	17	73.9	17	68.0	
	III	0	0.0	0	0.0	
	IV	5	21.7	6	24.0	
	V	1	4.3	2	8.0	
Class-III	I	1	2.2	2	6.1	0.003
	I'	0	0.0	0	0.0	
	II	39	86.7	19	57.6	
	III	1	2.2	0	0.0	
	IV	2	4.4	12	36.4	
	V	2	4.4	0	0.0	

## Data Availability

The datasets used and/or analyzed during the current study are available from the corresponding author.
